# Palmitate-induced autophagy liberates monounsaturated fatty acids and increases *Agrp* expression in hypothalamic cells

**DOI:** 10.1080/19768354.2019.1696407

**Published:** 2019-12-01

**Authors:** Seokjae Park, Tae Seok Oh, Seolsong Kim, Eun-Kyoung Kim

**Affiliations:** a Department of Brain and Cognitive Sciences, Daegu Gyeongbuk Institute of Science and Technology, Daegu, Republic of Korea; b Neurometabolomics Research Center, Daegu Gyeongbuk Institute of Science and Technology, Daegu, Republic of Korea

**Keywords:** Hypothalamic free fatty acids, autophagy, lipid droplet, *Agrp*, metabolomics

## Abstract

Fatty acids regulate food intake, although the exact mechanism remains unknown. Emerging evidence suggests that intracellular free fatty acids generated by starvation-induced autophagy regulate food intake. Starvation for 6 h elevated fatty acids such as palmitate, oleate, arachidonate, eicosatrienoate, and docosahexaenoate in the mouse serum. Among them, palmitate induced lipophagy, an autophagic degradation of cellular lipid droplets, in agouti-related peptide (*Agrp*)-expressing hypothalamic cells. Palmitate-induced lipophagy increased both *Agrp* expression and the contents of monounsaturated fatty acids such as palmitoleate, oleate, and (E)-9-octadecanoate, whereas these effects were blunted by autophagy deficiency. These findings support the role of free fatty acids in hypothalamic autophagy that regulates the appetite by changing the expression of orexigenic neuropeptides.

## Introduction

Accumulating evidence suggests that hypothalamic lipid metabolism regulates food intake and energy balance in the etiology of metabolic diseases (Lam et al. 2005; Lopez et al. 2005). Hypothalamic sensing of free fatty acids (FFAs) has been studied for years. Excess FFAs result in hypothalamic inflammation and dysfunction of the insulin and leptin signalling pathways, leading to impaired hypothalamic neuropeptide modulation and thereby increasing food intake (Benoit et al. 2009; Fick et al. 2011). Starvation increases the levels of FFAs in blood (Dole 1956; Sprangers et al. 2001; Rosen and Spiegelman 2006). Circulating FFAs are a primary fuel source in most tissues during starvation and they are also transported through the blood–brain barrier (Spector 1988), which may play a role in the appetite regulation. Fasting-induced circulating FFAs are rapidly taken up by a variety of cells including hypothalamic neurons and esterified to triglycerides within lipid droplets (LDs). Neuronal availability of FFAs has been proposed to regulate food intake in the hypothalamus (Lam et al. 2005; Andrews et al. 2008). Nevertheless, direct evidence to explain the regulatory mechanism of appetite by FFAs is insufficient. Administration of FFAs via intracerebroventricular (i.c.v.) injection directly regulates food intake. I.c.v. infusion of oleate decreases food intake as a result of a reduction in neuropeptide Y (*Npy*) mRNA and an increase in proopiomelanocortin (*Pomc*) neuron excitability (Obici et al. 2002; Jo et al. 2009; Schwinkendorf et al. 2011). In contrast, others have reported that i.c.v. infusion of palmitate shows orexigenic effects (Benoit et al. 2009; Milanski et al. 2009). These results imply distinct regulation of food intake depending on the type of FFA.

Autophagy can provide energetically essential components such as free fatty acids by breaking down intracellular organelles. Lipophagy is a type of selective autophagy that degrades cellular LDs and liberates FFAs, and serves as an important pathway for lipid metabolism (Singh et al. 2009; Singh and Cuervo 2012; Wang 2016; Schulze et al. 2017; Kim et al. 2019). Endogenous FFAs generated by starvation-induced hypothalamic lipophagy promote the expression of agouti-related peptide (*Agrp*), an orexigenic neuropeptide that stimulates food intake (Kaushik et al. 2011; Singh 2011; Liu and Czaja 2013). However, the link between intracellular FFAs liberated by lipophagy and *Agrp* expression is still unclear. In this study, we identified the types of FFAs that induce lipophagy and performed metabolomics analysis of FFAs generated by lipophagy that upregulates *Agrp* expression.

## Materials and methods

### Cell culture

The embryonic mouse hypothalamic mHypoE-N41 (N41; Cellutions Biosystems Inc., CLU121) cells were maintained in DMEM (Sigma) with 10% fetal bovine serum (Hyclone Laboratories Inc.) and 1% penicillin/streptomycin (Hyclone Laboratories Inc.) at 37°C.

### Animals

Male C57BL/6 mice were purchased from Koatech and housed (one per cage) in individually ventilated cages under a 12-h light/dark cycle (lights on from 6:00–18:00) in a temperature- and humidity-controlled room with *ad libitum* access to water and normal diet (LabDiet, Inc.). To measure blood glucose and fatty acids, the mice were fasted for 3, 6, and 12 h before the beginning of the dark period.

### Antibodies and chemical reagents

Target proteins were probed with the following antibodies: ATG5 and GAPDH (Cell Signaling Technology), LC3 (Sigma). For autophagy inhibition, cells were treated with E64d (Calbiochem) and pepstatin A (Calbiochem). Rapamycin (Sigma) was used to induce autophagy. E64d, pepstatin A, and rapamycin were dissolved in dimethyl sulfoxide (Sigma).

### Lipid droplet staining by boron-dipyrromethene

Intracellular LDs were stained using boron-dipyrromethene (BODIPY) 493/503 (Thermo Scientific). Cells were fixed with 4% formaldehyde in PBS, rinsed with PBS, and incubated with a 1:1000 dilution of a BODIPY stock (1 mg/ml in ethanol) in PBS for 15 min; nuclei were visualized by Hoechst 33342.

### siRNA transfection

N41 cells were seeded in 6-well plates and transfected with ON-TARGETplus mouse siRNA composed of 4 different siRNAs. Scrambled siRNAs (100 nM; Dharmacon) or *Atg5* siRNAs (100 nM; Dharmacon) were transfected using Lipofectamine 3000 (Invitrogen) for 48 h.

### Generation of CRISPR/Cas9-mediated *Atg5* knockout cells

The guide RNA for the *Atg5* gene inactivation (CACGTTTCCCACTTGCCTAGTGG; reverse frame) was designed by and purchased from ToolGen. N41 cells were transfected with Cas9 and gRNA plasmids (1:5 ratio) using TurboFect (Thermo Scientific). Homogenous *Atg5* knockout was achieved by selection using 1 mg/ml hygromycin (InvivoGen, ant-hg-1) at 24 h after transfection, followed by subculture in fresh DMEM.

### Fatty acid treatment

Sodium palmitate (Sigma), sodium oleate (Sigma), and arachidonate (Sigma) were dissolved in deionized water with heating. A mixture of the dissolved FAs (1 part) and DMEM (9 part) with 5% bovine serum albumin (Sigma) was added to culture media in a desired concentration.

### Fractionation of the nucleus and cytosol

Fractionation of the nucleus and cytosol was performed using nuclear and cytoplasmic extraction reagents (Thermo Scientific) following the manufacturer’s instructions. The nuclear pellet was used for GC-MS/MS analysis.

### Immunoblot analysis

Cells were lysed in lysis buffer (Lee et al. 2016). Lysates were resolved on SDS-polyacrylamide gels and blotted onto PVDF membranes (Millipore) for 35 min at 20 V in transfer buffer (25 mM Tris base, 192 mM glycine, 10% methanol, adjust to pH 7.4). The membranes were blocked with 5% skim milk for 1 h and incubated with appropriate primary antibodies for 1 h at room temperature or at 4°C overnight. After 3 washes with TBST buffer (20 mM Tris, 125 mM NaCl, 0.1% Tween 20), the membrane was incubated with appropriate HRP-linked secondary antibody (anti-mouse: CST; anti-rabbit: Thermo Scientific) and visualized by using ECL solutions (Thermo Scientific) according to the manufacturer’s instructions. Band intensities were measured and quantified using ImageJ software.

### Quantitative real-time PCR

Total RNA from cells or brain tissues was isolated using Trizol reagent (Invitrogen). The RNA pellet was dissolved in nuclease-free water (Promega), and total RNA concentration was determined using a NanoDrop spectrophotometer (Thermo Scientific). Total RNA, reaction buffer, and GoScript Reverse Transcriptase (Promega) were mixed in a total volume of 20 μl, and reverse transcription was carried out in a thermal cycler (Bio-Rad) at 25°C for 5 min, 42°C for 60 min, and 70°C for 15 min. Real-time PCR was performed with a SYBR Green PCR kit (TaKaRa Biotechnology) in a qPCR machine (Bio-Rad) for 40 cycles (95°C for 10 s, 60°C for 30 s). Relative mRNA level of *Agrp* (forward, 5′-CTGCAGACCGAGCAGAAGA-3′; reverse, 5′-TGCGACTACAGAGGTTCGTG-3′) was determined by the delta-delta Ct method and normalized to that of *Gapdh* (forward, 5′-ATCACTGCCACCCAGAAGAC-3′; reverse, 5′-ACACTTGGGGGTAGGAACA-3).

### Free fatty acid analysis

Serum (50 μl) and cells (5.0 × 10^5^) were sonicated with cold methanol:chloroform:water, 2:1:1 (v/v/v) containing an internal standard (20 nM D2-oleate). Samples were centrifuged at 4°C for 10 min at 12,000 × *g*. Organic phases were dried in a TurboVap evaporator (Biotage). Samples were reconstituted in hexane with 0.5 M KOH-MeOH and incubated at 25°C for 10 min. BCl_3_-MeOH (12% w/w) was then added. The samples were heated at 70°C for 10 min, cooled on ice for 5 min, and then hexane:water, 2:1 (v/v) were added. Samples were vortexed. The supernatants were transferred into autosampler vials. Fatty acid methyl esters (FAMEs) were analysed using a gas chromatograph – triple quadrupole tandem mass spectrometer (GC-QQQ-MS/MS; an Agilent 7890A series GC system coupled with an Agilent 7000C QQQ MS/MS). An Ultra HP-5 ms capillary column (30 m ×  0.25 μm, i.d. 0.25 μm film thickness, Agilent J&W Scientific) was used. The instrument temperature was set as follows: inlet, 250°C; transfer line, 290°C; ion source, 230°C; quadrupoles, 150°C. The capillary voltage was set at 70 eV. The gradient was run at a flow rate of 1.2 ml He/min, 4°C/min with 50°C up to 240°C. Detected FAMEs were quantified using MassHunter software (Agilent Technologies).

### Statistical analysis

All data are shown as mean ± SEM. Statistical significance was determined by Student *t* test using built-in software in GraphPad Prism 7. *p* values of <0.05 were considered statistically significant.

## Results

### Acute starvation alters serum free fatty acid profiles in mice

**Figure 1.**
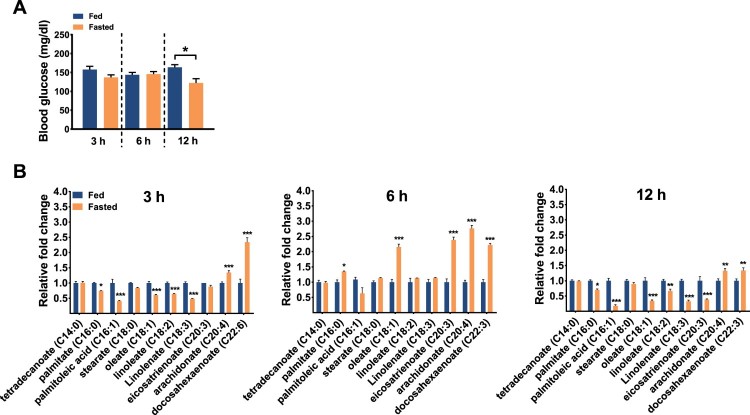
Changes in blood glucose level and serum free fatty acid profiles by acute starvation in mice. (A) Changes in blood glucose levels and (B) serum free fatty acid levels were measured in mice fasted for 3, 6, or 12 h. Data are mean ± SEM. *p < 0.05, **p < 0.01, and ***p < 0.001.

To identify specific FFAs that have a potential to generate a hunger signal in response to acute starvation, mice were fed or fasted for 3, 6, and 12 h from the beginning of the dark period. In accordance with previous reports showing lower glucose levels in plasma (Maeda et al. 2004; Andrikopoulos et al. 2008) and serum (Geisler et al. 2016) of fasted mice, blood glucose level was significantly lower in mice fasted for 12 h, but not at earlier time points (Figure 1A). However, serum FFA profiles fluctuated among the 3 time points. Arachidonate and docosahexaenoate showed higher levels in the fasted groups at all 3 time points compared to the fed groups. At 6 h, palmitate, oleate, eicosatrenoate, arachidonate, and docosahexaenoate were increased in the fasted groups. These FFAs were reduced after 12–h fasting (Figure 1B). These results suggest that some FFAs released by starvation may act as a hunger signal during acute fasting.

### SFA but not MUFA or PUFA induce autophagy in hypothalamic cells

**Figure 2.**
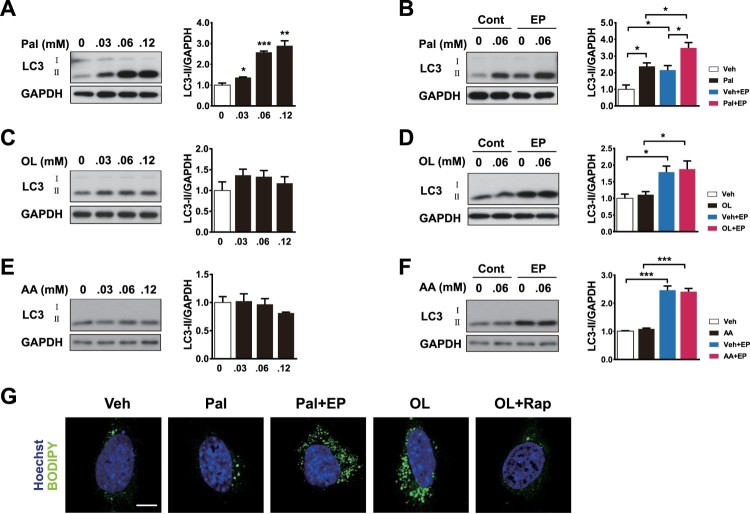
Palmitate but not oleate or arachidonate induces autophagy. (A) N41 cells were treated with palmitate (Pal) for 4 h at the indicated concentrations. The levels of LC3-II and GAPDH were determined by immunoblotting. (B) Treatment of palmitate-treated N41 cells with lysosomal protease inhibitors (EP), E64d (10 μg/ml) and pepstatin A (PepA: 10 μg/ml), for 4 h confirmed the increase in autophagy flux induced by palmitate. (C) Cells were treated with oleate (OL) under the same conditions and (D) autophagy flux was confirmed by EP treatment. (E) Cells were treated with arachidonate (AA) under the same conditions and (F) autophagy flux was confirmed by using EP. (G) Lipid droplets were stained with the fluorescent dye BODIPY in N41 cells treated with palmitate (Pal: 0.06 mM) or oleate (OL: 0.06 mM) with or without EP or rapamycin (Rapa), respectively, for 4 h. Nuclei were visualized by Hoechst. Scale bar: 10 μm. Data are mean ± SEM. *p < 0.05, **p < 0.01, and ***p < 0.001.

To identify whether autophagy is induced by saturated fatty acids (SFA), monounsaturated fatty acids (MUFA), or polyunsaturated fatty acids (PUFA) in hypothalamic cells, orexigenic neuropeptide – expressing N41 cells were treated with these FFAs. Palmitate, a type of SFA, elevated the levels of LC3-II in a dose-dependent manner (Figure 2A). Co-treatment with palmitate and lysosomal inhibitors (EP: E64d and pepstatin A) further increased the levels of LC3-II, indicating that the increases in LC3-II induced by palmitate reflect an increased autophagy flux (Figure 2B). In contrast, oleate (a MUFA) and arachidonate (a PUFA) did not alter LC3-II levels in N41 cells (Figure 2C–F). Inhibition of lysosomal activity with oleate and arachidonic acid treatment led to no further changes in LC3-II levels, confirming that oleate and arachidonate do not induce autophagy in N41 cells (Figure 2D and F).

### Palmitate induces lipophagy by degrading LDs in hypothalamic cells

To trace the changes in LDs induced by treatment with FFAs, we analysed the LDs by BODIPY staining in hypothalamic cells treated with palmitate or oleate in the presence of pharmacological reagents for regulating autophagy. EP, an autophagy inhibitor, was treated to hypothalamic cells with palmitate and rapamycin, an autophagy activator, was treated with oleate. Palmitate did not considerably affect LD staining, but in the presence of EP, LDs were accumulated because of autophagy inhibition (Figure 2G). In contrast, oleate-induced accumulation of LDs was reduced by rapamycin (Figure 2G). Taken together, these results suggest that palmitate but not MUFAs induces lipophagy by degrading LDs in the hypothalamic cells.

### Lipophagy regulation leads to changes in *Agrp* expression in the presence of FFAs

**Figure 3.**
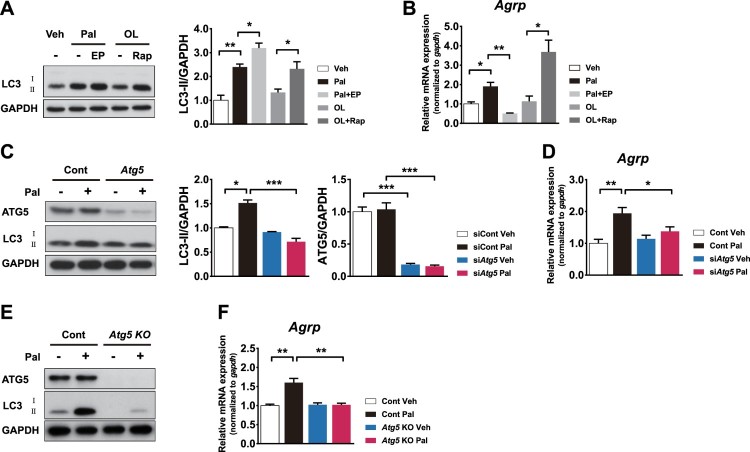
Autophagy regulation in the presence of palmitate leads to changes in *Agrp* expression. (A) LC3-II levels were analysed in N41 cells treated with palmitate (Pal: 0.06 mM) or oleate (OL: 0.06 mM) with or without lysosomal protease inhibitors (EP: E64d [10 μg/ml] and pepstatin A [PepA: 10 μg/ml]) and rapamycin (Rapa), respectively, for 4 h. (B) *Agrp* mRNA levels were analysed under the same conditions. (C) *Atg5* was knocked down using siRNAs and ATG5, LC3, and GAPDH were detected by immunoblotting in N41 cells treated with or without palmitate. (D) *Agrp* mRNA levels were analysed under the same conditions. (E) *Atg*5 knockout (*Atg5* KO) was conducted in N41 cells by the CRISPR/Cas9 system, and Atg5 and GAPDH were detected by immunoblotting. (F) The levels of *Agrp* mRNA in *Atg5* KO N41 cells with or without palmitate were measured by quantitative real-time PCR. Data are mean ± SEM. *p < 0.05, **p < 0.01.

Orexigenic neuropeptide expression was assessed in hypothalamic cells treated with palmitate and EP or oleate and rapamycin. LC3 immunoblots showed accumulation of LC3-II in N41 cells treated either with palmitate and EP, or with oleate and rapamycin, suggesting autophagy inhibition and induction was successful, respectively (Figure 3A). Under the same conditions, palmitate increased *Agrp* expression (Figure 3B). Notably, palmitate-induced *Agrp* expression was dramatically decreased by autophagy inhibition. In contrast, oleate failed to induce *Agrp* expression, but its expression was significantly increased when autophagy was induced by rapamycin (Figure 3B). To corroborate the effects of pharmacological modulation of autophagy, we genetically inhibited the autophagy-related gene 5 (*Atg5*) by small interfering RNAs (siRNAs; knockdown) or by the clustered regularly interspaced short palindromic repeat-cas system (CRISPR-Cas9; knockout). Palmitate failed to induce autophagy and *Agrp* expression under *Atg5-*knockdown or *Atg5-*knockout conditions (Figure 3C–F). Taken together, these results suggest that palmitate-induce lipophagy increases *Agrp* expression.

### Increases in MUFAs by palmitate are blunted in *Atg5* knockout

**Figure 4.**
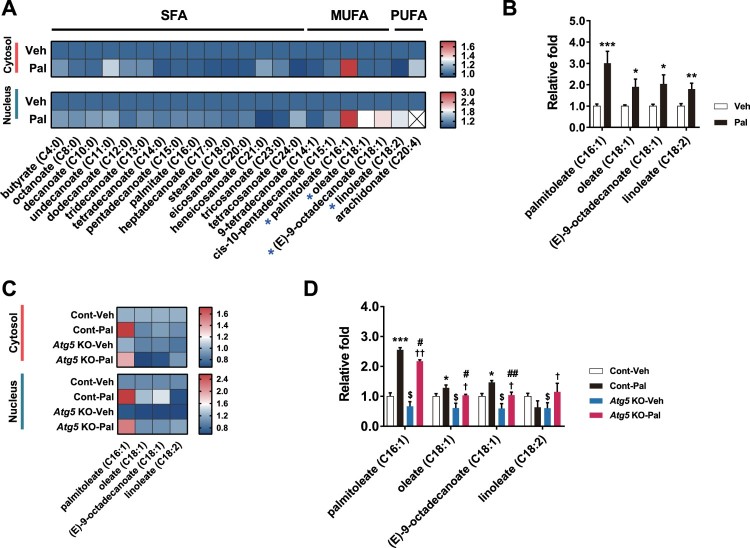
Lipophagy regulates contents of fatty acids in the cytosol and nucleus. (A) Heatmap of cytosolic/nuclear free fatty acids analysed by targeted GC-MS/MS metabolomics of normal hypothalamic cells treated with palmitate. (B) Palmitate elevates MUFAs (palmitoleate, oleate, (E)-9-octadecanoate) and PUFA (linoleate) in the nucleus. (C) Heatmap of cytosolic and nuclear MUFAs and PUFA analysed by targeted GC-MS/MS metabolomics of *Atg5* KO N41 cells treated with palmitate. (D) Increases in MUFAs induced by palmitate are blunted in *Atg5* KO cells. Data are mean ± SEM. Cont, control (no *Atg5* KO); Pal, palmitate; Veh, vehicle. *p < 0.05, **p < 0.01, ***p < 0.001 for Cont-Veh vs. Cont-Pal; ^$^*p* < 0.05 for Cont-Veh vs. *ATG5* KO-Veh; ^†^*p* < 0.05; ^††^*p* < 0.01 for *Atg5* KO-Veh vs. *Atg5* KO-Pal; #*p* < 0.05, ##*p* < 0.01 for Cont-Pal vs. *Atg5* KO-Pal.

To identify FFAs generated by palmitate-induced lipophagy, we analysed the FFA profiles by gas chromatography–triple-quadrupole tandem mass spectrometry (GC-QQQ-MS/MS) in *Atg5*-knockout cells treated with palmitate. Palmitoleate levels were significantly increased by palmitate in the cytosol of hypothalamic cells (Figure 4A and B). In addition, the levels of palmitoleate, oleate, (E)-9-octadecanoate, and linoleate were significantly increased in the nucleus. Interestingly, the increases in MUFAs (palmitoleate, oleate and (E)-9-octadecanoate) by palmitate were significantly blunted in *Atg5*-knockout cells (Figure 4C and D).

## Discussion

In the present study, we demonstrated that lipophagy induced in hypothalamic cells by palmitate, a specific type of SFAs, degrades cytosolic LDs into intracellular FFAs and increases *Agrp* expression in hypothalamic cells. The metabolomics analysis indicated an increase in MUFAs liberated from LDs by lipophagy. *Atg5* deficiency study confirmed that these changes are autophagy-dependent.

Intracellular FFAs generated by autophagy may have multiple functions. In general, FFAs are metabolized by catabolic processes, such as mitochondrial β-oxidation, and anabolic processes, which produce biologically important molecules such as phospholipids, second messengers, and local hormones (Wakil and Abu-Elheiga 2009; Wrighton 2015). On the other hand, autophagy may provide a source of signalling molecules by degrading LDs under low-nutrient conditions to drive appetite. In hypothalamic GT1-7 cells, an increase in fatty acid content by starvation-induced autophagy regulates lipid metabolism and Agrp expression (Kaushik et al. 2011). Interestingly, FFAs liberated by lipophagy in hypothalamic tanycytes increase ATP production and reduce the expression of orexigenic neuropeptides in mice with high fat diet – induced obesity (Kim et al. 2019). Our results show that inhibition of autophagy leads to accumulation of intracellular LDs accompanied by attenuation of a palmitate-induced increase in *Agrp* gene expression. In this regard, derivatives of intracellular FFAs may play a role in the transcriptional regulation of neuropeptides responsible for appetite control, and autophagy could play a critical role in this regulation by providing signalling molecules.

Palmitate reportedly has orexigenic effects, and these effects were blunted by autophagy inhibition (Benoit et al. 2009; Milanski et al. 2009; Kaushik et al. 2011). However, these orexigenic effects are controversial; one study reported that i.c.v. infusion of palmitate had no effect on 24-h food intake compared to control (Schwinkendorf et al. 2011), whereas another study reported no acute effect on food intake in fasted mice but blunted anorexigenic action of leptin (Kleinridders et al. 2009). Food intake is inhibited by central administration of MUFAs (Obici et al. 2002; Jo et al. 2009; Schwinkendorf et al. 2011) and by intravenous infusion of oleate (Oh et al. 2016). Ingestion of palmitoleate reduces appetite by activating the CCK signalling pathway (Nunes and Rafacho 2017), but the role and mechanism of appetite regulation by these FFAs in the hypothalamus have not been determined. It seems that diverse physiological environments determine the roles of different types of FFAs in food intake.

Our results suggest that increases in MUFAs by palmitate-induced lipophagy in the hypothalamic nucleus may be involved in transcriptional regulation of *Agrp* expression. Unsaturated fatty acids play a role in the interaction with transcription factors. Oleate and PUFAs including ω-3, ω-6 bind to the ligand of peroxisome proliferator activated receptors (PPARs) and activate gene transcription in the liver (Varga et al. 2011; Jump et al. 2013). In the brain, docosahexaenoate binding to retinoid X receptor (RXR) regulates lipid metabolism and cholesterol transport (de Urquiza et al. 2000). Therefore, further studies would be of interest to investigate whether MUFAs in the nucleus interact with transcriptional factors to regulate *Agrp* transcription.
